# Painting a portrait: Analysis of national health survey data for cancer genetic counseling

**DOI:** 10.1002/cam4.1864

**Published:** 2019-02-07

**Authors:** Monica H. Stamp, Ora K. Gordon, Christopher P. Childers, Kimberly K. Childers

**Affiliations:** ^1^ Center for Clinical Genetics and Genomics Providence St. Joseph Health Los Angeles California; ^2^ Division of Genetics Providence John Wayne Cancer Institute Santa Monica California; ^3^ Department of Surgery David Geffen School of Medicine at UCLA Los Angeles California; ^4^ Department of Health Policy and Management Fielding School of Public Health at UCLA Los Angeles California

**Keywords:** genetic counseling, health care disparities, hereditary cancer, national survey, population health

## Abstract

**Background:**

Despite a growing body of literature describing the geographic and sociodemographic distribution of cancer genetic testing, work focused on these domains in cancer genetic counseling is limited. Research describing the epidemiology of cancer genetic counseling has mainly focused on isolated populations, a single gender (women) and a single condition (hereditary breast and ovarian cancer). Study findings to date are contradictory, making it unclear what, if any, disparities in receipt of cancer genetic counseling exist.

**Methods:**

Utilizing the 2015 National Health Interview Survey (NHIS)—a cross‐sectional, in person interview survey collecting self‐reported health data for the US population—geographic and sociodemographic factors were compared between those receiving genetic counseling and the national sample. Bivariate analysis and subsequent multivariable logistic regression were performed with stratification by cancer status (affected/unaffected). Reason for (eg, doctor recommended) and focus of (eg, breast/ovarian) genetic counseling were also assessed. To generate nationally representative estimates, all analyses were adjusted for survey weights.

**Results:**

An estimated 4.8 million individuals in the United States had cancer genetic counseling. On bivariate analysis, there were significant differences in proportions undergoing genetic counseling by sex, race/ethnicity, insurance, citizenship, education, age, and cancer status (*P* < 0.01). After adjustment, however, only female sex (Odds Ratio [OR]: 1.78 [95% CI: 1.18‐2.67]) remained a significant predictor of genetic counseling among the affected. Among the unaffected, female sex (OR: 1.70 [1.30‐2.21]), non‐Hispanic black race (OR: 1.44 [1.02‐2.05], reference: non‐Hispanic white), graduate education (OR: 1.76 [1.03‐2.98], reference: less than high school), and age (OR: 1.06 [1.01‐1.11]) predicted higher rates of genetic counseling. An estimated 2.1 million individuals have undergone genetic counseling focused on breast/ovarian cancer, 1.3 million on colorectal cancer, and 1.4 million on “other” cancers. Of those receiving genetic counseling focused on breast/ovarian cancer, 3% were male and 97% female (breast cancer alone‐4% male, 96% female); for colorectal cancer, 49% male and 51% female, and for “other” cancers, 60% male and 40% female. The majority of individuals receiving genetic counseling reported they did so because their doctor recommended it (66%), with smaller proportions describing self (12%), family (10%), or media (5%) influences as the primary reason.

**Conclusion:**

This is the first study to depict the sociodemographic and geographic distribution of cancer genetic counseling at the national level. Despite perceived disparities in access, cancer genetic counseling in the United States appears to be accessed by individuals of diverse racial/ethnic backgrounds, with various insurance coverage and educational levels, and across a broad range of ages and geographic regions. The only sociodemographic factor that independently predicted receipt of genetic counseling across both the affected and unaffected population was sex. With physician recommendation as the predominant driver for counseling, targeting physician education, and awareness is crucial to utilization.

## 1 INTRODUCTION

At least 5%‐15% of cancer diagnoses in the United States are due to inherited cancer syndromes.^1^, ^2^, ^3^, ^4^ Common examples are hereditary breast and ovarian cancer syndrome (HBOC; eg, *BRCA1/2*) and Lynch syndrome. Population carrier rates for these pathogenic variants are high, ranging from 1/40 to 1/400 for *BRCA1/2* (depending on ethnic background) and 1/300 to 1/400 for Lynch syndrome.^5^, ^6^, ^7^

Genetic counseling is critical to identify patients at risk for inherited cancer syndromes, facilitate genetic testing, and guide management decisions. The National Society of Genetic Counselors defines genetic counseling as integrating “Interpretation of family and medical histories to assess the chance of disease occurrence or recurrence. Education about inheritance, testing, management, prevention, resources, and research. Counseling to promote informed choices and adaptation to the risk or condition”.^8^ This process often involves one or more visits with a genetic counselor (board certified allied health professional), specially trained nurse, or physician, and involves shared decision‐making between the patient and provider. Genetic counseling may involve but is a distinct process from, genetic testing. The US Preventive Services Task Force and the National Comprehensive Cancer Network recommend genetic counseling for cancer risk assessment in those meeting select personal and/or family history criteria, including diagnosis of breast or colorectal cancers at or under age 50, any diagnosis of ovarian cancer, or multiple affected relatives. Further, many insurance companies now require formal genetic counseling by a certified genetics provider prior to genetic testing.^9^, ^10^, ^11^

Despite the recommendation for genetic counseling, limited data exist regarding the geographic and sociodemographic distribution of cancer genetic counseling in the United States. The literature on genetic testing is more robust, but this population may be distinct from those receiving genetic counseling.^12^, ^13^, ^14^, ^15^, ^16^, ^17^, ^18^, ^19^, ^20^, ^21^, ^22^ Previous literature related to cancer genetic counseling has been narrow in scope, often focused on only one segment of the population—such as women, the affected, or a single inherited syndrome (HBOC). Further, most previous studies have been confined to a single geographic region or health care system, limiting generalizability. Conclusions from regional research have been mixed in regards to the impact of race, age, education, and insurance on the receipt of genetic counseling.^21^, ^22^, ^23^, ^24^, ^25^, ^26^, ^27^ In fact, for any given covariate—such as race—studies have found evidence of both positive and negative associations. For example, a number of prior studies have demonstrated that women of black race are less likely to obtain genetic counseling than individuals of white race, but a recent study on women with newly diagnosed breast cancer actually found individuals of black race are more likely to obtain genetic counseling.^21^, ^22^, ^24^, ^26^ Similar conflicting findings have been found for age, education, and insurance status.^21^, ^22^, ^23^, ^24^, ^25^, ^26^, ^27^ While a number of prior studies have identified higher education to be associated with use of genetic counseling, the above study on women with newly diagnosed breast cancer found that education was not significant.^21^, ^23^, ^26^ That same study identified insurance type as a significant predictor of genetic counseling; however, another study on those with a family history of cancer demonstrated that insurance type was not associated with counseling use.^21^, ^26^ The reason for these highly disparate findings is unclear but may reflect sampling bias introduced by only assessing a limited population.

As the scope of genetic medicine expands, data regarding the distribution of cancer genetic counseling is imperative. Specifically, data reflecting rates of genetic counseling obtained by both men and women, with and without personal cancer histories, for indications beyond breast/ovarian cancer (ie, colorectal) is needed in order to fully understand national trends and identify disparities. This study uses a nationally representative sample to assess the geographic and sociodemographic distribution of cancer genetic counseling in the United States. Understanding this distribution can help guide future educational efforts, policy development, and service delivery modeling in genetic medicine.

## 2 MATERIALS AND METHODS

### 2.1 Data source and ethics review

This study utilized publicly available data from the 2015 National Health Interview Survey (NHIS). The NHIS is an annual cross‐sectional in person household interview survey conducted by the Centers for Disease Control to assess the health status of the United States noninstitutionalized civilian population.^28^ The survey has been conducted for over 50 years and serves as the primary source of health information to guide national health policy. Questions address a broad array of topics such as insurance status, disability, vaccinations, injuries, nutrition, medical conditions, and sociodemographics. Every 5 years, the survey asks detailed questions about cancer, family history, and genetic testing, the results of which are uploaded into an Adult Cancer File. Questions specific to genetic counseling were first added to this file in 2015. We extracted sociodemographic and geographic factors from two of the annual data files (Person, Sample Adult) and merged them with the Adult Cancer File.

The 2015 survey response rate was 79.7% and included 33 672 respondents. The survey utilizes complex multistage sampling incorporating geographic and building permit information. This sampling strategy is redesigned after every decennial census in order to ensure national representation. Further, minority populations, such as certain racial/ethnic groups as well as older individuals, are oversampled in order to improve the precision of estimates in these populations.^29^ Data are publicly available and de‐identified and therefore did not meet the definition of human participants research.

### 2.2 Variable definitions

The primary outcome of interest was receipt of genetic counseling for cancer. The stem for the genetic counseling questions was as follows: “These next questions refer to genetic counseling for cancer risk. We will ask about genetic testing for cancer risk in a few minutes. Genetic counseling involves a discussion with a specially trained health care provider about your family history of cancer and how likely you are to develop cancer. It may also include a discussion about whether genetic testing is right for you.” Respondents were then asked to state whether or not they had received genetic counseling for cancer risk. If affirmative, the respondent was then asked the main reason for genetic counseling (your doctor recommended it; you requested it; family member suggested it; you heard or read about it in the news; and, “other”) and the clinical focus of genetic counseling (breast cancer, ovarian cancer, colon or rectal cancer [“colorectal”], or another type of cancer [“other,” defined as any other cancer type outside of breast, ovarian, and colorectal]). Answers to the latter did not have to be mutually exclusive nor did they have to be concordant with personal or family history.

Sociodemographic factors were extracted for each observation, including sex (male, female), US Census region, race/ethnicity (NHIS does not capture Ashkenazi Jewish ancestry), insurance, citizenship, highest educational achievement, and age. To facilitate regression analysis, we generated mutually exclusive insurance categories with priority given to private insurance. For example, individuals with both Medicare and Private insurance would be classified as Private. When available, insurance information was verified with the individual's membership card. US citizenship was based on self‐reported birth in the United States or US territory, birth overseas to US parents, or naturalization. Highest educational achievement was converted to a categorical variable. We analyzed age as both a continuous and categorical variable. In the NHIS, age is censored at 85 years old to protect participant confidentiality. Finally, we extracted the cancer status of each observation, labeling individuals either as “affected” (personal history of any cancer) or as “unaffected” (no personal history of cancer).

### 2.3 Study aims

This study had four aims: (a) compare the geographic and sociodemographic distribution of those receiving genetic counseling to the broader US population, (b) analyze geographic and sociodemographic factors by cancer status (affected, unaffected), (c) describe the clinical focus of genetic counseling sessions (eg, colorectal cancer), and (d) assess the main reason for obtaining genetic counseling (eg, your doctor recommended it).

### 2.4 Analytic and statistical approach

To address the first and second aims, we generated descriptive data for the entire NHIS sample. We then repeated this analysis on the subset of individuals who had received genetic counseling and compared the counseled subset and the national sample using bivariate tests and multivariable logistic regression models. The regression model was stratified by cancer status (affected, unaffected). For the third and fourth aims, descriptive data were generated for the clinical focus (breast, ovarian, colorectal, and other) and reason for genetic counseling.

Statistical analysis was conducted using STATA v 15.1. All estimates were adjusted for complex survey weights following guidelines outlined by NHIS in order to generate national estimates.^28^ Bivariate comparisons were made using adjusted Wald tests. Regression models were run using complete case analysis. Standard errors were estimated using the linearized Taylor series method. Statistical significance was determined using two‐sided tests and a *P* value of 0.05.

## 3 RESULTS

### 3.1 The 2015 NHIS sample

The 2015 data set included 33 672 observations weighted to represent 242 500 657 adults (age 18 and older) in the noninstitutionalized civilian US population. Of these, 52% were female, and the majority were Non‐Hispanic white (66%), privately insured (66%), and US citizens (92%). Nine percent of individuals reported a personal history of cancer (Table 1).

**Table 1:** Geographic and sociodemographic features of those receiving genetic counseling, including comparisons with the national sample

		Whole sample		GC subsample		
	Unweighted N	33 672		706		
	Weighted(1) N	242 500 657		4 807 915		
		Prop (1)(2)	(SE)	Prop (1)(2)	(SE)	*P*‐value
Sex	Male	48%	(0.4%)	34%	(2.5%)	<0.001
	Female	52%	(0.4%)	66%	(2.5%)	
Region(3)	Northeast	17%	(0.4%)	19%	(2.2%)	NS
	Midwest	22%	(0.4%)	19%	(2.1%)	
	South	37%	(0.5%)	39%	(2.5%)	
	West	23%	(0.4%)	23%	(2.2%)	
Race/ethnicity	Hispanic	16%	(0.3%)	10%	(1.3%)	0.009
	Non‐Hispanic White	66%	(0.4%)	71%	(2.2%)	
	Non‐Hispanic Black	12%	(0.3%)	14%	(1.7%)	
	Non‐Hispanic Asian	6%	(0.2%)	5%	(1.1%)	
	Other	1%	(0.1%)	1%	(0.7%)	
Insurance(4)	Private	66%	(0.4%)	69%	(2.3%)	<0.001
	Medicaid/Other Public	11%	(0.3%)	10%	(1.4%)	
	Medicare	8%	(0.2%)	9%	(1.3%)	
	Other	5%	(0.2%)	8%	(1.4%)	
	Uninsured	10%	(0.3%)	4%	(0.9%)	
Citizenship	Citizen	92%	(0.3%)	95%	(1.0%)	<0.001
	Not Citizen	8%	(0.3%)	5%	(1.0%)	
Highest educational achievement	Less than HS	13%	(0.3%)	10%	(1.5%)	<0.001
	HS, GED, or some college	44%	(0.4%)	37%	(2.6%)	
	Associates	11%	(0.3%)	12%	(1.5%)	
	Bachelors	20%	(0.3%)	24%	(2.4%)	
	Graduate	12%	(0.3%)	17%	(2.0%)	
Age category	18‐25	14%	(0.4%)	7%	(1.9%)	<0.001
	26‐35	18%	(0.3%)	12%	(1.5%)	
	36‐45	17%	(0.3%)	14%	(1.7%)	
	46‐55	18%	(0.3%)	23%	(2.4%)	
	56‐65	16%	(0.3%)	20%	(1.9%)	
	66‐75	11%	(0.2%)	15%	(1.7%)	
	76‐85+	7%	(0.2%)	9%	(1.4%)	
History of cancer	Yes	9%	(0.2%)	32%	(2.3%)	<0.001
	No	91%	(0.2%)	68%	(2.3%)	

GC, genetic counseling; GED, General Education Diploma; HS, High School; IHS, Indian Health Service; NCHS, National Center for Health Statistics; NHIS, National Health Interview Survey; SCHIP, State Children's Health Insurance Program; US.

(1) Estimates adjusted for complex survey design and including weights.

(2) Covariate data missing for <1% of observations; proportions estimated based on non‐missing.

(3) Regions consistent with US Census definition.

(4) Mutually exclusive categories with priority given to private insurance; Medicaid category includes SCHIP and other state‐sponsored insurance programs; "Other" includes Military coverage (eg Tricare), IHS, and other governmental coverage.

Data Source: NCHS, National Health Interview Survey.

### 3.2 Distribution of genetic counseling and bivariate analysis

Two percent of individuals (n = 706) reported genetic counseling for cancer risk, weighted to represent 4 807 915 US adults.

On bivariate analysis, all sociodemographic factors except geographic region were significantly different between the overall sample and the genetic counseling subsample (all *P* < 0.01). There appeared to be overrepresentation of females, those with a personal history of cancer (affected), those with bachelors and graduate level degrees, and older individuals in the subsample. In contrast, Hispanics, the uninsured, noncitizens, and younger individuals appeared to be underrepresented (Table 1).

### 3.3 Regression modeling of genetic counseling by cancer status

On logistic regression, Hispanic race, citizenship, and insurance were no longer associated with receipt of genetic counseling for neither the unaffected nor the affected. Among the unaffected, female sex (OR: 1.70 [1.30‐2.21]), non‐Hispanic black race (OR: 1.44 [1.02‐2.05], reference: non‐Hispanic white), graduate education (OR: 1.76 [1.03‐2.98], reference: less than HS) and older age (OR: 1.06 [1.01‐1.11]) predicted higher odds of genetic counseling (Table 2). In the affected population, only female sex (OR: 1.78 [1.18‐2.67]) remained a significant predictor of genetic counseling (Table 2).

**Table 2:** Regression analysis analyzing the effect of geographic and sociodemographic factors on receipt of cancer genetic counseling, including stratification by cancer status

		Unaffected			Affected		
		Odds ratio	*P*‐value	95% CI	Odds ratio	*P*‐value	95% CI
	Unweighted N	27 804			3047		
	Weighted N(1)	200 846 984			19 522 113		
Sex	Male	1.0 (Reference)			1.0 (Reference)		
	Female	1.70	0.00	(1.30‐2.21)	1.78	0.01	(1.18‐2.67)
Region(2)	Northeast	1.0 (Reference)			1.0 (Reference)		
	Midwest	0.83	0.36	(0.55‐1.25)	0.61	0.17	(0.31‐1.24)
	South	0.96	0.82	(0.67‐1.37)	0.74	0.33	(0.41‐1.35)
	West	0.96	0.85	(0.66‐1.42)	0.87	0.65	(0.48‐1.59)
Self‐reported race/ethnicity	Hispanic	0.74	0.15	(0.50‐1.11)	1.68	0.10	(0.90‐3.12)
	Non‐Hispanic White	1.0 (Reference)			1.0 (Reference)		
	Non‐Hispanic Black	1.44	0.04	(1.02‐2.05)	1.25	0.49	(0.66‐2.38)
	Non‐Hispanic Asian	0.76	0.28	(0.47‐1.25)	1.71	0.43	(0.46‐6.42)
	Other	1.90	0.27	(0.61‐5.97)	1.37	0.70	(0.28‐6.83)
Insurance(3)	Private	1.0 (Reference)			1.0 (Reference)		
	Medicaid/Other Public	1.01	0.96	(0.66‐1.55)	0.77	0.48	(0.37‐1.59)
	Medicare	0.92	0.75	(0.54‐1.55)	0.63	0.11	(0.36‐1.10)
	Other	1.72	0.02	(1.09‐2.71)	0.70	0.26	(0.37‐1.30)
	Uninsured	0.63	0.09	(0.37‐1.07)	0.50	0.11	(0.21‐1.17)
Citizenship	Citizen	1.0 (Reference)			1.0 (Reference)		
	Not Citizen	0.76	0.32	(0.44‐1.31)	1.76	0.22	(0.71‐4.34)
Highest educational achievement	Less than HS	1.0 (Reference)			1.0 (Reference)		
	HS, GED, or some college	1.11	0.62	(0.73‐1.70)	0.67	0.25	(0.34‐1.33)
	Associates	1.32	0.30	(0.78‐2.21)	0.67	0.34	(0.29‐1.53)
	Bachelors	1.62	0.06	(0.98‐2.70)	1.01	0.98	(0.47‐2.16)
	Graduate	1.76	0.04	(1.03‐2.98)	0.92	0.84	(0.42‐2.01)
Age		1.06	0.02	(1.01‐1.11)	1.01	0.79	(0.93‐1.10)
Age^2		1.00	0.04	(1.00‐1.00)	1.00	0.49	(1.00‐1.00)

Complete case analysis; 91.6% of unaffected and 92.6% of affected individuals with complete data.

GED, General Education Diploma; HS, High School; IHS, Indian Health Service; NCHS, National Center for Health Statistics; NHIS, National Health Interview Survey; SCHIP, State Children's Health Insurance Program; US.

(1) Estimates adjusted for complex survey design and including weights.

(2) Regions consistent with US Census definition.

(3) Mutually exclusive categories with priority given to private insurance; Medicaid category includes SCHIP and other state‐sponsored insurance programs; "Other" includes Military coverage (eg, Tricare), IHS, and other governmental coverage.

Data Source: NCHS, National Health Interview Survey.

### 3.4 Clinical focus of and reason for genetic counseling

An estimated 2.1 million individuals have undergone genetic counseling focused on breast/ovarian cancer, 1.3 million on colorectal cancer, and 1.4 million on “other” cancers. Of those receiving genetic counseling for breast cancer, 4% were male and 96% were female. For colorectal cancer, 49% were male and 51% were female. For “other” cancer, 60% were male and 40% were female (Table 3).

**Table 3:** Clinical focus of genetic counseling, stratified by sex

	Breast/Ovarian		Breast Only		Ovarian Only		Colorectal		Other	
	Prop (1)	(SE)	Prop (1)	(SE)	Prop (1)	(SE)	Prop (1)	(SE)	Prop (1)	(SE)
Unweighted N	339		286		132		188		196	
Weighted N	2 142 625		1 820 121		748 069		1 258 928		1 398 158	
Men	3%	(1.3%)	4%	(1.5%)	NA		49%	(4.9%)	60%	(4.1%)
Women	97%	(1.3%)	96%	(1.5%)			51%	(4.9%)	40%	(4.1%)

(1) Estimates adjusted for complex survey design and including weights.

Data Source: NCHS, National Health Interview Survey.

The majority of individuals receiving genetic counseling did so because their doctor recommended it (65%), with smaller proportions reporting self (12%), family (10%), or media (5%) influences (Table 4).

**Table 4:** Reason for obtaining genetic counseling

		Prop (1)(2)	(SE)
	Unweighted N	703	
	Weighted N	4 791 218	
Main reason for getting genetic counseling?	Doctor recommended	66%	(3.3%)
	You requested it	12%	(1.5%)
	Family member suggested it	10%	(1.8%)
	Heard or read about it in news	5%	(2.6%)
	Other	7%	(1.6%)

(1) Estimates adjusted for complex survey design and including weights.

(2) Three individuals with missing data (reflected in sample size decrease from 706); proportions estimated for non‐missing.

Data Source: NCHS, National Health Interview Survey.

## 4 DISCUSSION

An estimated 4.8 million individuals in the United States have received cancer genetic counseling. This is the first study to describe the sociodemographic and geographic distribution of cancer genetic counseling at the national level, including both men and women, affected and unaffected, and a variety of clinical indications (breast/ovarian, colorectal, and other). While a number of factors initially appeared to be associated with receipt of counseling—such as race, education, and insurance—these disparities disappeared after appropriate stratification and multivariable adjustment. The only sociodemographic factor that independently predicted receipt of genetic counseling across both the affected and unaffected population was sex.

Prior research on genetic counseling inequality has focused largely on isolated health systems or geographic areas, women, and a single clinical condition (hereditary breast/ovarian cancer).^21^, ^22^, ^23^, ^24^, ^25^, ^26^, ^27^ Further, the literature that does exist is highly contradictory, with disparities noted in age, race/ethnicity, education level, and insurance.^21^, ^22^, ^23^, ^24^, ^25^, ^26^, ^27^ Again, the reason for these highly disparate findings is unclear but may reflect the problem of sampling bias introduced by only assessing a limited population.

In broadening the study population beyond a single sex, region, and clinical indication, the findings of this study provide a more comprehensive understanding of the discrepancies related to genetic counseling access. The most notable disparity in this study is sex, with females obtaining genetic counseling twice as often as their male counterparts across both affected and unaffected groups. This translates to an estimated 1.5 million more women obtaining genetic counseling than men. In the affected population, with breast/ovarian cancer diagnoses as the primary reason for testing, this gap is well explained. However, in the unaffected population, carrier rates in men and women should be identical (per autosomal dominant inheritance). This important finding has been overlooked by previous studies that have only focused on women and HBOC, despite the importance of genetic counseling for men. One hypothesis for this disparity may be that men approach preventive care differently than women.^30^ However, our study did not identify a counseling disparity in men and women obtaining testing for colorectal and “other” cancers. If differences in health care utilization were the primary reason for this discrepancy, then one would anticipate equally low rates of genetic counseling in men across cancer types. This finding parallels that of a recent genetic testing study that also found low rates of testing among men for breast/ovarian cancer risk, but found no testing variation in colorectal and other cancers.^19^ The finding that only breast/ovarian counseling rates are disparate in men may suggest instead a failure of identifying and referring men at risk for HBOC, or a perceived lack of relevance for counseling in men for HBOC. It is important to note that men with *BRCA1/2* pathogenic variants, for example, are not only at risk for male breast cancer, but also melanoma, pancreatic, and prostate cancers.^31^, ^32^, ^33^ Furthermore, male BRCA carriers who develop cancer are diagnosed with higher grade and later stage disease for both breast and prostate cancers.^31^, ^32^ Recent studies on men with prostate cancer have noted that men with *BRCA2* pathogenic variants have a higher mortality compared to their *BRCA2*‐negative counterparts.^32^ Lastly, identifying male carriers is invaluable to inform risk assessment for future generations (daughters and sons). Thus, identifying men at risk is crucial to aid in early cancer detection and prevention for men and their family members.

An important aspect of our study was the stratification of individuals by cancer status. In contrast to affected individuals, where the only independent predictor was sex, additional gaps existed in the unaffected population including race/ethnicity, age, and education. These findings parallel those in recent study on the same NHIS data set that was confined to unaffected women and HBOC.^24^ Similar to our study, they found that education and age were significant predictors for genetic counseling; in contrast to our study, a significant association with race was not found.^24^ By expanding the population to both sexes and including all cancer indications, non‐Hispanic Black race/ethnicity was found to be a significant positive predictor of genetic counseling. The reason for this is unclear and has not been demonstrated before in the unaffected population. Perhaps earlier papers, which identified a significant disparity, have fueled increased awareness of and referral for genetic counseling in this population.

Higher education was a significant predictor of genetic counseling only in the unaffected population. It has been hypothesized that unaffected women with higher educational levels may be more likely to proactively discuss genetic testing with their primary care providers.^13^ Interestingly, education level was not significant in the affected population; this may be due to the fact that affected individuals are typically seen in an oncology setting where providers may initiate discussion of genetic counseling more readily compared to in the primary care setting. Lastly, only in the unaffected population was age a significant predictor of genetic counseling. There are several possible explanations for this age discrepancy; it may be that family history presents with age as cancers develop in older adult relatives.^34^ Another explanation could be that the majority of cancer risk occurs in middle to late adulthood, prompting genetic counseling at that age. However, this age skewing may also represent under‐recognition of the importance of genetic counseling for younger adults, as management for high‐risk conditions such as HBOC and Lynch syndrome begin as early as age 20‐25.^9^

In this study, an estimated 2.1 million individuals of the 4.8 million who received genetic counseling reported receiving counseling for future risk of breast/ovarian cancer, compared to 1.3 million for colorectal cancer, and 1.4 million for “other” cancers. No previous national study has published the rates of genetic counseling across cancer types. Given that research suggests *BRCA1/2* and Lynch syndrome gene pathogenic variants have a similar prevalence in the US population, this may represent a discrepancy, with under‐recognition of Lynch syndrome compared to HBOC.^5^, ^6^, ^7^ Regardless, the fact that over half of the population has obtained genetic counseling for colorectal and “other” cancers highlights the importance of expanding studies of genetic counseling beyond breast/ovarian cancer to truly appreciate population‐level disparities. This study serves as a benchmark for future research.

The finding that the majority of individuals receiving genetic counseling report that their doctor recommended the service highlights the continued value of genetic counseling as an ancillary clinical service. It also demonstrates the importance of maintaining strong physician relationships to ensure continued utilization of genetic counseling. However, individuals are also seeking genetic counseling through other mechanisms—such as self‐referral, family recommendation, and hearing or reading about genetic counseling in the news. Given the rising market and publicity for direct to consumer genetic testing, individuals may turn to these options for genetic information in the future. Ensuring that these individuals can obtain genetic counseling will be imperative to aid in results interpretation and management. Therefore, continued improvement of access to care and community outreach is vital to ensure all individuals who may benefit from genetic counseling know about and have access to the service.

There are several limitations to this study. First, NHIS collects primarily self‐reported data without external validation. Thus, responses are limited by recall bias related to cancer history, receipt of genetic counseling, and focus of as well as reason for counseling. Despite using the most recent 2015 data, there has been a rapid evolution in cancer genetics, including pretest genetic counseling mandates by insurance companies, increased awareness of genetic counseling and testing in general, and broader, more inclusive referral guidelines related to indications for genetic counseling. Therefore, this data may not fully represent the current landscape of those accessing genetic counseling. However, it provides a strong foundation for future research to identify trends in counseling over time. Lastly, NHIS compiles all responses on the reason for genetic counseling as “other” if counseling was not related to breast, ovarian, or colorectal cancer. This prevents analysis of genetic counseling related to risk of cancer outside of these three organs, which may be important as individuals begin to access genetic counseling related to concern for other cancer types.

Future research should move beyond the sociodemographic distribution of those obtaining genetic counseling to focus on overall access to and availability of genetic counseling services. As current data suggest low counseling and testing rates, assessing what barriers to testing exist via genetic counseling, as well as mechanisms to improve genetic testing rates through genetic counseling, are imperative.^32^, ^35^, ^36^ Further, given the emerging importance of additional cancer types, such as prostate and pancreatic, as indications of hereditary cancer syndromes, a more robust survey design is needed to include these specifically among possible reasons for genetic counseling. Updates should include specific information regarding indication for counseling related to personal and/or family history, as well as intent of counseling, to help identify whether disparities exist in specific cancer predisposition syndromes and/or cancer types.

In conclusion, an estimated 4.8 million individuals in the United States have undergone cancer genetic counseling. With the exception of sex, cancer genetic counseling appears to be accessed by individuals of diverse racial/ethnic backgrounds, with various insurance coverage and educational levels, and across a broad range of ages and geographic regions in the United States. National education efforts are needed to address the gender disparity, and thus enable early cancer detection and prevention in men. This study highlights the need for continued engagement with physician partners as the most frequent referral sources for cancer genetic counseling. Depicting the national distribution of cancer genetic counseling can provide insight into growing opportunities for genetics providers to offer leadership in care delivery for hereditary cancer risk assessment.

## CONFLICT OF INTEREST

None declared.

## AUTHOR CONTRIBUTIONS

All Authors: conceptualization, data curation, investigation, methodology, writing—original draft, and writing—review and editing; Chris Childers: formal analysis.
